# A Comparison of IgG Index and Oligoclonal Band in the Cerebrospinal Fluid for Differentiating between RRMS and NMOSD

**DOI:** 10.3390/brainsci12010069

**Published:** 2021-12-31

**Authors:** Bo Chen, Dai-Shi Tian, Bi-Tao Bu

**Affiliations:** Department of Neurology, Tongji Hospital, Tongji Medical College, Huazhong University of Science and Technology, Wuhan 430030, China; bochen@tjh.tjmu.edu.cn (B.C.); bubitao@tjh.tjmu.edu.cn (B.-T.B.)

**Keywords:** IgG index, oligoclonal band, relapsing–remitting multiple sclerosis, neuromyelitis optica spectrum disorder, onset age

## Abstract

As the oligoclonal band in the cerebrospinal fluid (CSF-OCB) in predicting relapsing-remitting multiple sclerosis (RRMS) is less sensitive in Asian populations than that in westerners, it remains elusive whether the IgG index could serve as an alternative. The purpose of this study was to compare these two methods of differentiating between RRMS and neuromyelitis optica spectrum disorder (NMOSD) in Chinese patients. A total of 171 patients (81 RRMS and 90 NMOSD) were retrospectively recruited, of whom 82 (56 RRMS and 26 NMOSD) received the CSF-OCB testing additionally. When the onset age was ≤38.5 years, IgG index with the threshold of 0.67 had a significant agreement (k = 0.4, *p* < 0.001) with the diagnosis while CSF-OCB failed to discriminate (k = 0.1, *p* = 0.578). However, when the onset age was >38.5 years, both IgG index with the threshold of 0.8 and CSF-OCB were moderately consistent with the diagnosis (both k > 0.4, *p* < 0.05). In total, our optimized algorithm had the sensitivity, specificity, and predictive accuracy of 0.778, slightly outperforming the CSF-OCB model. Accordingly, a combination of the onset age and IgG index could serve as an alternative to CSF-OCB for differentiating between RRMS and NMOSD in Chinese patients.

## 1. Introduction

The presence of “oligoclonal bands” (OCBs) in the cerebrospinal fluid (CSF) has been identified as a key immunodiagnostic biomarker for multiple sclerosis (MS), detected in about 95% of clinically definite cases in western countries [1,2]. CSF-OCBs are the soluble clonal immunoglobulins resulting from the intrathecal immune response against antigens that are still not completely known. Although the CSF-OCB does not imply dissemination in time, it can substitute for the requirement for demonstration of this measure [3], accelerating the diagnosis for MS without waiting for an additional attack. However, isoelectric focusing (IEF) gel electrophoresis, a frequently used sensitive and accurate technique for detecting CSF-OCB [4], is not always available or affordable in many rural areas. More importantly, in patients with MS, CSF-OCB-positive cases in many Asian countries account for a much lower proportion than those in western countries [5,6,7], suggesting that the sensitivity of this laboratory marker may vary between races. This, on the other hand, would also lead to a delayed diagnosis in a considerable proportion of Asian patients. In this scenario, the IgG index, a traditional and expedient method measuring the intrathecal IgG synthesis, seems to be an alternative.

Although previous studies have noted that the IgG index is less sensitive than the CSF-OCB by IEF in predicting MS [1,4,8,9] and an elevated IgG index does not necessarily herald the presence of CSF-OCB [3], these results are based on westerners and a fixed threshold, e.g., 0.7, for all the individuals. In fact, the IgG index may also be influenced by other factors apart from diseases, e.g., age. This is because theoretically, the age-dependent dysfunction or delayed recovery of the blood–brain barrier may facilitate autoreactive B cells that evade tolerance checkpoints and aberrantly break tolerance to autoantigens to migrate from the periphery to the central nervous system (CNS), where they possibly differentiate into antibody-secreting cells (ASCs) (i.e., plasmablasts and plasma cells) [10] and contribute to an elevated IgG index in certain conditions. Moreover, as the onset age is an important classifier between CNS-demyelinating dysimmunities, e.g., patients with MS frequently had younger ages at onset than those with neuromyelitis optica spectrum disorder (NMOSD) [11,12,13,14], a combination of this measure and the IgG index may improve the diagnostic accuracy to some extent.

Accordingly, in this study, we aim to investigate the clinical significance of the IgG index in predicting Chinese patients with MS through systemically comparing it with CSF-OCB, used for differentiating the common CNS-demyelinating conditions based on age stratification, and hope to provide a clue for the preliminary diagnostic aid in the neuroimmunology clinic.

## 2. Materials and Methods

### 2.1. Participants

Patients’ data were retrospectively reviewed from the electronic medical record system in the department of neurology in Tongji Hospital, Tongji Medical College, Huazhong University of Science and Technology, between December 2019 and October 2021. Only participants who fulfilled the following criteria were included: diagnosis of relapsing–remitting MS (RRMS) according to the 2017 revisions of the McDonald criteria [3] and aquaporin-4 antibody (AQP4-Ab) positive NMOSD based on the 2015 international consensus [15]; available CSF data with IgG index. Cases who received immunosuppressive treatment or disease-modifying therapies (DMTs) for at least 6 consecutive months closely before lumbar puncture (LP) were excluded from our study, along with those who were administered with rescue therapies (pulsed glucocorticoids, plasma exchange, and intravenous immunoglobulins) within one month before LP. AQP4-Ab was detected by cell-based assay (CBA).

### 2.2. IgG Index and CSF-OCB

All recruited patients received the tests for IgG index within one month of the clinical attack, some of whom additionally received the CSF-OCB detection performed by IEF (SEBIA HYDRASYS), with both CSF and blood samples in the two testings obtained at the same time. The IgG index was detected using a rate immunonephelometry technique (SIEMENS BN-II) and calculated as the quotient of the QIgG (CSF-to-serum IgG ratio) and Qalb (CSF-to-serum albumin ratio). Detection of CSF-OCBs was defined as positive if patterns 2 or 3 were present [4,16].

### 2.3. Statistical Analyses

Mann–Whitney test was used for continuous distributions to compare between groups, with Fisher’s exact test for nominal data. Bivariate and partial correlations, as well as their significance, were performed by Spearman’s rank correlation analyses. Cohen’s kappa (k) was used to assess the agreement of the IgG index and CSF-OCB with the diagnosis. A k < 0 refers to poor agreement, 0.01–0.2 slight, 0.21–0.4 fair, 0.41–0.6 moderate, 0.61–0.8 substantial, and 0.81–1.0 approximately perfect agreement. The optimal cutoff value or threshold associated with the outcome (diagnosis) was determined by receiver operating characteristic (ROC) curve analysis. The significance of the stratified variable in the crosstab was assessed by Cochran and Mantel–Haenszel (CMH) tests. All data were analyzed using IBM SPSS 23 software, with a 2-sided *p* < 0.05 considered significant.

## 3. Results

A total of 171 patients (81 with RRMS and 90 with AQP4-Ab NMOSD) were recruited, and their clinical laboratory profiles are summarized in Table 1, with 108 and 82 participants who additionally received the tests for the serum complement component (C3 and C4) and CSF-OCB, respectively. The sex ratio was similar between the two groups (*p* = 0.22), while the patients with RRMS had a significantly lower onset age and age at LP than those with AQP4-Ab NMOSD (both *p* < 0.001). Additionally, the IgG index and the number of positive CSF-OCB in the RRMS group were markedly higher than those in the NMOSD counterpart (both *p* < 0.001), whereas a relatively elevated Qalb was observed in the latter group (*p* = 0.005). Notably, neither of the complement components C3 nor C4 in the serum was statistically different between groups (both *p* > 0.1).

To clearly demonstrate the correlations between variables, Spearman’s correlation analysis was used and showed that the diagnosis was significantly associated with the age at onset, with the correlation coefficient of 0.54, followed by the age at LP (0.516), the number of positive CSF-OCB (−0.418), and the IgG index (−0.411). This suggested that age, especially at the onset, could serve as the preliminary classifier between RRMS and AQP4-Ab NMOSD.

### 3.1. Identification of the Optimal Cutoff Values between Groups by ROC Curve Analysis

A preliminary classification was built based on the onset age performed by ROC curve analysis, which revealed that 38.5 years could serve as the optimal cutoff value in differentiating between the two diseases, with the sensitivity of 0.741 and specificity of 0.8 (*p* < 0.001). Then, in the early onset age (onset age ≤ 38.5 y) group, the optimal threshold of the IgG index for prediction was 0.67 (sensitivity: 0.783 and specificity: 0.667, *p* = 0.001); while in its late-onset age (onset age >38.5 y) counterpart, this figure was 0.8 (sensitivity: 0.762 and specificity: 0.806, *p* < 0.001), i.e., an elevated IgG index was defined as a value > 0.67 when the onset age was ≤38.5 y or that ≥0.8 when the age was >38.5 y.

### 3.2. Comparison of the IgG Index and CSF-OCB for Differentiating between RRMS and AQP4-Ab NMOSD Based on the Stratification of the Onset Age

Crosstabs based on the stratification of the onset age were built to compare the differences between IgG index and CSF-OCB in the diagnostic prediction (Table 2). In the early onset age group, the CSF-OCB failed to differentiate between RRMS and AQP4-Ab NMOSD (k = 0.1, *p* = 0.578), whereas it could achieve moderate agreement with the final diagnosis in the late-onset age group (k = 0.43, *p* = 0.015), with significant CMH tests (both *p* < 0.01). By comparison, the IgG index could predict the diagnosis in both early and late-onset age groups with a fair-to-moderate agreement (both k ≥ 0.4 and *p* < 0.001), and the CMH tests were also significant (both *p* < 0.001). Notably, when patients with different onset ages were pooled, both IgG index and CSF-OCB could well classify the two conditions (both *p* < 0.001), with a relatively higher agreement observed when the IgG index served as the classifier (IgG index vs. CSF-OCB: 0.56 vs. 0.4).

In the early onset age group, although CSF-OCB had a slightly higher positive predictive value (PPV) than IgG index (0.935 vs. 0.887), the latter outperformed the former in sensitivity, specificity, negative predictive value (NPV), and predictive accuracy (PA). However, with the increase in age at onset (>38.5 y), in the scheme in which CSF-OCB served as the discriminator, the sensitivity and PPV decreased, while the specificity, NPV, and PA were elevated, with similar trends also observed in the scheme in which IgG index was employed as a distinguisher. Meanwhile, the IgG index algorithm had a markedly high NPV (0.921) yet a lower PPV (0.533) than its CSF-OCB counterpart. Nonetheless, in the pooled group, concerning all the assessments except PPV, the IgG index had an improved level of performance, compared with CSF-OCB, in differentiating between the two diseases.

Notably, if the patients with RRMS who had only one clinical attack were excluded, the optimal cutoff value of the onset age remained 38.5 y, but the CSF-OCB failed to predict the diseases in either early or late-onset age group (both k < 0.3, *p* > 0.1), while the IgG index still could (both k > 0.3, *p* < 0.01) (Supplementary Table S1). Moreover, in the populations with early and late-onset age, the CSF-OCB and IgG index were in moderate (k = 0.6, *p* < 0.001) and substantial (k = 0.64, *p* < 0.001) agreement with each other, respectively.

Interestingly, if the age at LP served as the preliminary classifier, with the optimal threshold of 44.5 y, almost the same results as those achieved by the onset age were observed (Supplementary Table S2). However, if the threshold of the IgG index was set at 0.7, regardless of age, the specificity and predictive accuracy would drop to 0.6 and 0.69, respectively, while the sensitivity was similar (0.79). Again, if the presence of intrathecal IgG synthesis was defined according to Reiber’s hyperbolic function [17], the agreement was almost the same as that achieved by the IgG index with the threshold of 0.7 but lower than that in our scheme, in both early and later-onset age groups (Supplementary Table S3).

### 3.3. Establishment of a Discriminative Model

**Figure 1:**
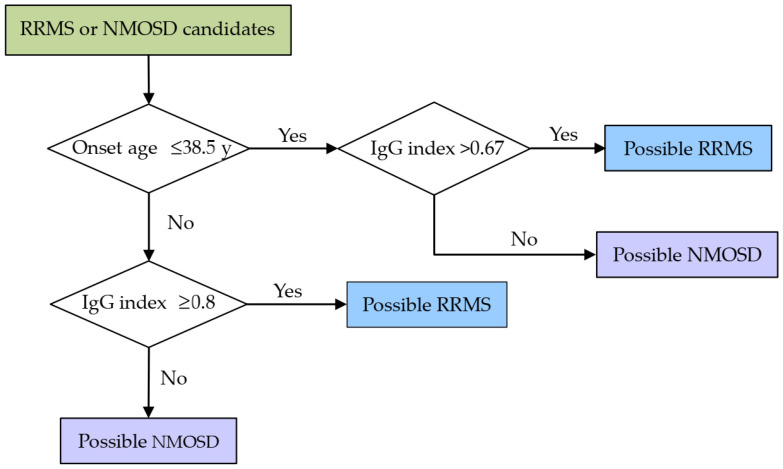
A two-step algorithm for discriminating between RRMS and NMOSD based on the onset age and IgG index. Abbreviations: RRMS = relapsing–remitting multiple sclerosis; NMOSD = neuromyelitis optica spectrum disorder.

According to these results above, a simple discriminative practice scheme based on the onset age and IgG index was constructed (Figure 1), with both the sensitivity and specificity of 0.778, slightly higher than those based on CSF-OCB (sensitivity: 0.679 and specificity: 0.769). Interestingly, when the onset age and IgG index were included as continuous independent variables in the binary logistic analysis, the overall percentage correct was 78.4%, almost the same as ours (77.8%).

### 3.4. Sex-Related Differences in CSF Analysis between RRMS and NMOSD

As the blood–CSF barrier permeability may vary between males and females, we further investigated this difference and found that female sex was correlated with a lower Qalb and total protein, as well as albumin level, in CSF of both MS and NMOSD groups (Table 3). However, when the onset age was controlled, the female sex was still borderline associated with lower CSF protein levels in the MS group, but these markers did not vary between sexes in the NMOSD group (Supplementary Table S4), with the similar results observed when the age at LP was controlled.

## 4. Discussion

In this study, we compared the IgG index and CSF-OCB for distinguishing RRMS from AQP4-Ab NMOSD and revealed that the IgG index could better discriminate between the two diseases based on the stratification of the onset age and different cutoff values. When the age at onset was no more than 38.5 y, the IgG index with the threshold of 0.67 could achieve fair agreement (k = 0.4, *p* < 0.001) with the diagnosis, while the CSF-OCB failed (k = 0.1, *p* = 0.578). However, when the patient was older than 38.5 y at onset, both IgG index with the cutoff value of 0.8 and CSF-OCB could predict the final diagnosis with moderate consistency (both k > 0.4, *p* < 0.05). These results suggest that it may not be appropriate to classify Chinese patients with early onset ages by the presence of CSF-OCB, whereas the predictive values of the IgG index and CSF-OCB were similar in those with late-onset ages. On the other hand, these also imply that the IgG index still has a role in discriminating between RRMS and NMOSD. In centers where AQP4 antibody (by CBA) and CSF-OCB (by IEF) assays are not available or in cases where these tests are not affordable for candidate patients with RRMS or NMOSD, our practical scheme could be applied as a preliminary diagnostic aid or may provide diagnostic clues as to which patients should be detected especially for antibodies.

Traditionally, a threshold for the elevated IgG index for all the individuals to predict the potential intrathecal IgG synthesis is commonly defined, regardless of age, and in many laboratories in China, including our center, this value is usually set at 0.7. Nevertheless, we found that this scheme had a lower specificity and predictive accuracy than our algorithm, with a similar sensitivity between the two. Further analysis revealed that the IgG index was borderline associated with the age at onset (correlation coefficient: 0.198, *p* = 0.062) in patients with NMOSD, while this association was statistically absent in those with RRMS (correlation coefficient: 0.045, *p* = 0.689) (Supplementary Table S4), suggesting that the correlation of the onset age and the IgG index may vary between the two diseases. Generally, Qalb is considered a measure of the blood–CSF barrier function and is age dependent [18]. Although IgG is larger than albumin in molecular size [17], QIgG, to a lesser extent, could have similar properties to Qalb in the absence of intrathecal IgG synthesis. These findings were evinced by our study—namely, that both of the Qalb and QIgG were positively correlated with the onset age or age at LP in both RRMS and NMOSD group (Supplementary Table S4, both *p* < 0.001), implying an age-dependent, attenuated blood–CSF barrier function. This dysfunction may subsequently facilitate the passive transfer of the albumin and immunoglobulin as well as the migration of immune cells, including the antibody-secreting cells (ASCs), from the periphery to the CNS and lay a foundation for the intrathecal immunoglobulin synthesis, which may partially account for the elevated IgG index with the increased age in patients with NMOSD, leading to a rise in the optimal cutoff value of IgG index (0.8) between NMOSD and RRMS when the candidates aged more than 38.5 y. In this context, the schemes that simply employed the presence of intrathecal IgG synthesis as the distinguisher would lead to the low specificity. By contrast, our optimized algorithms maximized the discrimination by defining different thresholds of the IgG index based on the stratification of the onset age, regardless of the presence of intrathecally synthesized IgG, and demonstrated a potentially improved discriminative ability (Supplementary Table S3). However, it is still elusive why the IgG index was not associated with the onset age or age at LP in RRMS herein (Supplementary Table S4). In fact, although intrathecally synthesized immunoglobulins and the presence of CSF-OCBs could imply a diagnosis of MS, the underlying mechanisms and their pathogenic significance remain intriguing. Antibody deposition can be observed histologically in MS lesions [19] and rituximab, a B-cell-depleting agent, has been proved to be effective in limiting the disease activity [20,21,22,23], suggesting that B-cell-driven humoral dysimmunity may also play a critical role in the pathogenesis of MS. Nevertheless, the lack of specific pathogenic antibodies implies that other functions of B cells apart from antibody secretion, including antigen presentation to Th cells and cytokine production, may be more important [24]. On the other hand, the onset age is an optimal classifier between RRMS and NMOSD, with the highest association with the diagnosis in our study (correlation coefficient: 0.54, *p* < 0.001, Table 1). Therefore, a predictive scheme based on the IgG index and the age at onset is probably rational, which is also proved by significant CMH tests.

Notably, an increased IgG index did not necessarily herald the presence of CSF-OCB because detection of CSF-OCB was considered positive if patterns 2 or 3 were present [16], albeit both of the two markers were highly correlated in the whether RRMS or NMOSD group (both *p* < 0.05, Supplementary Table S4). Since the presence of CSF-OCB is not bound to conclude the diagnosis of RRMS or exclusion of NMOSD, especially in Asian countries, where the positivity of CSF-OCB in the population with RRMS is not as high as that in western ones [5,6,7], a scheme that IgG index discriminates between the two diseases through predicting the presence of CSF-OCB would obviously lead to decreased sensitivity and specificity. However, theoretically, in extreme cases in which the blood–CSF barrier is fully disrupted, the maximum of the IgG index would be 1 in the absence of intrathecal IgG synthesis, i.e., an IgG index ≥ 1 can definitely predict a positive CSF-OCB. Moreover, as CSF-OCB can substitute for the requirement for the dissemination in time, accelerating the diagnosis of MS in a considerable number of patients with only one clinical attack, the inclusion of these patients may elevate the IgG index in the RRMS group to some extent, given the high correlation between the IgG index and CSF-OCB. When they were excluded, CSF-OCB failed to classify the diseases correctly in either early or later onset age group (both k < 0.3, *p* > 0.1) while the IgG index still could (both k > 0.3, *p* < 0.01) (Supplementary Table S1), outperforming CSF-OCB.

Interestingly, although patients with NMOSD had a significantly higher Qalb value than those with RRMS (Table 1), Qalb did not differ when the onset age (*p* = 0.864) or the age at LP (*p* = 0.983) was controlled in the partial correlation analysis (Supplementary Table S5), suggesting that the blood–CSF barrier function may be similarly influenced between the two diseases in the case of the same age, and thus, the age-dependent, attenuated barrier function seems to be the main contributor to this difference in Qalb. On the other hand, this could also imply that the two distinct immune responses are likely to exert a negligible discrepancy on the barrier function. Or, Qalb may not be sensitive enough to perceive the local blood–CSF barrier breakdown, assuming that this disruption is indeed remarkable in patients with NMOSD.

Both the onset age and age at LP herein were significantly correlated with the diagnosis (*p* < 0.001, Table 1), yet the former seemed superior, as it had a slightly higher correlation coefficient (onset age vs. age at LP: 0.54 vs. 0.516). Theoretically, the age at LP is additionally associated with the disease duration, which could dilute its predictive effect, as both of the patients with RRMS and NMOSD are vulnerable to relapse and can have long durations, particularly when the durations vary significantly. When the age at LP instead of the onset age served as the preliminary classifier in our model, its optimal cutoff value was 44.5 y. This algorithm would achieve the same diagnostic accuracy (0.778) for NMOSD and RRMS candidates in total as the scheme in our study, possibly owing to the similar median durations between the two conditions (RRMS vs. NMOSD: 3 y vs. 3.5 y, Table 1). Both discriminative schemes are statistically acceptable, whereas, in clinical practice, the onset age often tends to serve as the preliminary classifier due to its relatively higher correlation with the diagnosis.

The sex-related differences in blood–CSF barrier permeability were previously observed in a CSF analysis between hospital and general populations [25], between patients with MS, other inflammatory neurological disorders, and non-inflammatory [26], as well as between those with schizophreniform and affective psychosis [27]. Although females are generally vulnerable to dysimmunities, male patients had a significantly higher total protein in CSF than females across all ages [28]. In line with this finding, we also noted that female patients had lower protein levels, including total protein, IgG, and albumin, in CSF than males, in both RRMS and NMOSD groups (Table 3). Nonetheless, further analysis revealed that these sex-related differences also varied between diseases when the onset age (Supplementary Table S4) or the age at LP was controlled, suggesting that the age may exert a different effect on this sex-related variance in blood–CSF barrier permeability between NMOSD and RRMS.

Although the vasculocentric deposition of immunoglobulin and activated complement components was observed in the NMOSD lesions [29,30,31], we noted that the concentrations of complement component C3 and C4 in the serum did not significantly differ between patients with NMOSD and RRMS, with similar findings seen in the IgG level in the serum and CSF (Table 1), suggesting that the depletion of these immune mediators resulting from the different CNS immune responses is likely to be inappreciable for an individual.

Our study was limited by the relatively small number of the patients, especially those with NMOSD who received CSF-OCB testing, which is possibly attributed to the selection bias in clinical practice and may lead to a potentially incorrect estimate of the diagnostic value of this testing method. In addition, admittedly, dichotomizing a continuous variable is not the optimal solution statistically [32] but has practical significance, which could help the clinicians draw a rapid and preliminary impression on the candidate patients. Nevertheless, we further validated that our results were in agreement with those produced by the binary logistic regression model, suggesting that this algorithm is rational. In future studies, there is an urging need for research with more patients, in multiple centers, in other Asian countries, and more parameters included.

## 5. Conclusions

In summary, the IgG index may still have a role in discriminating between RRMS and AQP4-Ab NMOSD, as CSF-OCB in China is not as sensitive as that in western countries. Different thresholds defined based on the stratification of the onset age or age at LP can improve the predictive value of the IgG index and help achieve, at least, the non-inferior, or even superior, accuracy and sensitivity to the CSF-OCB herein. Accordingly, this optimized scheme may be applied as an alternative in cases where CSF-OCB testing by IEF is unavailable or affordable. Notably, although the diagnosis of MS has been facilitated by the advancement of MRI imaging, it remains a great challenge to differentiate it from the MS mimics, especially in those with only spinal or optic nerve injuries or one clinical attack, due to lack of a specific antibody similar to AQP4-Ab in NMOSD. Comprehensively collecting and assessing the data from a candidate patient is required before a probable diagnosis is drawn. Occasionally, time will tell.

## Figures and Tables

**Table 1:** Clinical laboratory profiles of the participants.

Variables	RRMS	AQP4-Ab NMOSD	*p*	Correlation Coefficient ^▲^
No. of participants	81	90		
No. of females (%)	57 (70.3)	71 (78.9)	0.22	−0.098
Age at onset, median (range), year	30 (14–68)	48.5 (18–72)	<0.001	0.540
Age at LP, median (range), year	33 (14–68)	52 (18–72)	<0.001	0.516
CSF				
Total protein, median, mg/L (range)	326 (160–1167)	381.5 (185–2883)	0.029	0.168
Albumin, median, mg/L (range)	182 (95–711)	219 (87–1790)	0.023	0.175
IgG, median, mg/L (range)	43.4 (12.2–155)	38.05 (9.3–448)	0.319	−0.076
Serum				
Albumin, median, g/L (range)	42.1 (28.8–49.1)	39.75 (33.1–51.5)	<0.001	−0.279
IgG, median, g/L (range)	10.3 (4.7–21.1)	10.7 (4.1–35.7)	0.746	0.025
C3, g/L (range) ^#^	0.75 (0.56–1.21)	0.76 (0.55–1.22)	0.484	−0.068
C4, g/L (range) ^#^	0.18 (0.07–0.4)	0.17 (0.08–0.49)	0.797	−0.025
Qalb, median (range)	4.22 (2.05–18.37)	5.47 (2.28–49.31)	0.005	0.215
QIgG, median (range)	4.24 (1.04–14.83)	3.67 (1.12–24.62)	0.477	−0.055
IgG index, median (range)	0.87 (0.4–2.09)	0.66 (0.37–1.46)	<0.001	−0.411
No. of positive CSF-OCB (%) *	38 (67.9)	6 (23.1)	<0.001	−0.418

Abbreviations: RRMS = relapsing–remitting multiple sclerosis; AQP4-Ab NMOSD = aquaporin-4 antibody neuromyelitis optica spectrum disorder; LP = lumbar puncture; CSF = cerebrospinal fluid; C3 = complement component 3; C4 = complement component 4; Qalb = quotient of albumin in CSF and albumin in serum; QIgG = quotient of IgG in CSF and IgG in serum; CSF-OCB = oligoclonal band in CSF. # Data from 108 patients (41 with RRMS and 67 with AQP4-Ab NMOSD) were available. * Data from 82 patients (56 with RRMS and 26 with AQP4-Ab NMOSD) were available. ^▲^ Spearman’s correlation analysis: a positive value means the variable was associated with AQP4-Ab NMOSD, while a negative one indicates that the variable was correlated with RRMS.

**Table 2:** Comparison of the IgG index and CSF-OCB for differentiating between RRMS and AQP4-Ab NMOSD based on the stratification of the onset age.

Onset Age	Methods	Results	Diagnosis		k	*p*	Sen	Spe	PPV	NPV	PA
			RRMS	NMOSD							
≤38.5 y	CSF-OCB	−	12	2	0.10	0.578	0.707	0.5	0.935	0.143	0.689
		+	29	2							
>38.5 y		−	6	18	0.43	0.015	0.6	0.818	0.692	0.75	0.73
		+	9	4							
Total		−	18	20	0.40	<0.001	0.679	0.769	0.864	0.526	0.707
		+	38	6							
≤38.5 y	IgG index	− *	13	12	0.40	<0.001	0.783	0.667	0.887	0.48	0.756
		+	47	6							
>38.5 y		−	5	58	0.49	<0.001	0.762	0.806	0.533	0.921	0.796
		+	16	14							
Total		−	18	70	0.56	<0.001	0.778	0.778	0.759	0.795	0.778
		+	63	20							

* A “−” refers to an IgG index lower than the defined threshold while a “+” indicates a value greater than the defined threshold. Abbreviations: RRMS = relapsing–remitting multiple sclerosis; NMOSD = neuromyelitis optica spectrum disorder; CSF-OCB = oligoclonal band in the cerebrospinal fluid; Sen = sensitivity; Spe = specificity; PPV = positive predictive value; NPV = negative predictive value; PA = predictive accuracy.

**Table 3:** Spearman’s correlation analysis of the sex-related difference in the CSF protein levels between RRMS and NMOSD.

Diagnosis	Female	CSF-Tpro	CSF-Alb	CSF-IgG	Qalb	IgG Index
RRMS	Correlation coefficient	−0.218	−0.248	−0.115	−0.201	−0.16
	*p*	0.051	0.026	0.306	0.072	0.155
NMOSD	Correlation coefficient	−0.27	−0.275	−0.132	−0.253	0.057
	*p*	0.01	0.009	0.217	0.016	0.593

Abbreviations: RRMS = relapsing–remitting multiple sclerosis; NMOSD = neuromyelitis optica spectrum disorder; CSF = cerebrospinal fluid; Tpro = total protein; Alb = albumin; Qalb = quotient of albumin in CSF and IgG in serum; CSF-OCB = oligoclonal band in CSF.

## Data Availability

The data presented in this study are available on request from the corresponding author (D.S.T.). The data are not publicly available due to ethical restrictions.

## Supplementary Materials

The following are available online at https://www.mdpi.com/article/10.3390/brainsci12010069/s1, Table S1: Comparison of the IgG index and CSF-OCB for differentiating between RRMS patients with more than one clinical attack and patients suffering from AQP4-Ab NMOSD based on the stratification of the onset age, Table S2: Comparison of the IgG index and CSF-OCB for differentiating between patients with RRMS and AQP4-Ab NMOSD based on the stratification of the age at LP, Table S3: The agreement with diagnosis achieved by different methods; Table S4: Spearman’s bivariate correlation analysis between NMOSD and RRMS; Table S5: Partial correlation analyses between Qalb and diagnosis while controlling the onset age or age at LP.
