# Supplementary material for: A Comparison of IgG Index and Oligoclonal Band in the Cerebrospinal Fluid for Differentiating between RRMS and NMOSD
Source: Brain Sci. 2021 Dec 31;12(1):69. doi: 10.3390/brainsci12010069 (PMC8773790; doi:10.3390/brainsci12010069)
Supplement: Supplementary file 1 [file brainsci-12-00069-s001.zip › brainsci-1503233-supplementary.pdf]

Supplementary Table S1. Comparison of the IgG index and CSF-OCB for differentiating between RRMS patients with more than one clinical attack and patients suffering from AQP4-Ab NMOSD based on the stratification of the onset age.

**Onset age**	**Methods**	**Results**	**Diagnosis**		**κ**	***p***
			**RRMS**	**NMOSD**		
≤38.5y	CSF-OCB	−	12	2	0.027	> 0.99
		+	15	2		
>38.5y		−	6	18	0.286	0.121
		+	5	4		
Total		−	18	20	0.275	0.022
		+	20	6		
≤38.5y	IgG index#	− *	13	12	0.343	0.009
		+	33	6		
>38.5y		−	2	46	0.339	< 0.001
		+	15	26		
Total		−	15	58	0.391	< 0.001
		+	48	32		

# The optimal cutoff values for IgG index in differentiating between the two diseases were 0.67 when the onset age was <=38.5y and 0.75 when the onset age was >38.5y, respectively.
* A "−" refers to an IgG index lower than the defined threshold, while a "+" indicates a value greater than the defined threshold.
Abbreviations: RRMS = relapsing–remitting multiple sclerosis; NMOSD = neuromyelitis optica spectrum disorder; CSF-OCB = oligoclonal band in the cerebrospinal fluid.

Supplementary Table S2. Comparison of the IgG index and CSF-OCB for differentiating between patients with RRMS and AQP4-Ab NMOSD based on the stratification of the age at LP.

**Age at LP**	**Methods**	**Results**	**Diagnosis**		**κ**	***p***	**Sen**	**Spe**	**PPV**	**NPV**	**PA**
			**RRMS**	**NMOSD**							
≤44.5y	CSF-OCB	−	13	8	0.32	0.014	0.705	0.727	0.912	0.381	0.709
		+	31	3							
>44.5y		−	5	12	0.39	0.057	0.583	0.80	0.70	0.706	0.704
		+	7	3							
Total		−	18	20	0.40	< 0.001	0.679	0.769	0.864	0.526	0.707
		+	38	6							
≤44.5y	IgG index	−	14	23	0.47	< 0.001	0.785	0.697	0.836	0.622	0.755
		+	51	10							
>44.5y		−	4	47	0.51	< 0.001	0.75	0.825	0.545	0.922	0.808
		+	12	10							
Total		−	18	70	0.56	< 0.001	0.778	0.778	0.759	0.795	0.778
		+	63	20							

Abbreviations: RRMS = relapsing–remitting multiple sclerosis; NMOSD = neuromyelitis optica spectrum disorder; CSF-OCB = oligoclonal band in the cerebrospinal fluid; Sen =

sensitivity; Spe = specificity; PPV = positive predictive value; NPV = negative predictive value; PA = predictive accuracy.

Supplementary Table S3. The agreement with diagnosis achieved by different methods.

	**Onset age**	**κ**	***p***
Reiber's hyperbolic function*	≤38.5y	0.38	0.001
	>38.5y	0.32	< 0.001
	Total	0.40	< 0.001
IgG index with cutoff of 0.7	≤38.5y	0.36	0.002
	>38.5y	0.33	< 0.001
	Total	0.39	< 0.001
Our scheme	≤38.5y	0.40	< 0.001
	>38.5y	0.49	< 0.001
	Total	0.56	< 0.001

* Based on the calculation by Reiber's hyperbolic function, the upper limit of quantitative intrathecal IgG synthesis is determined by the following equation:

Qlim (IgG) = $0.93\sqrt{\text{Qalb}^2 + 6 \times 10^{-6}} - 1.7 \times 10^{-3}$

Therefore, the presence of intrathecal IgG synthesis was defined as the QIgG greater than Qlim (IgG).

Supplementary Table S4. Spearman's bivariate correlation analysis between NMOSD and RRMS.

**Diagnosis**	**Variables**	**Onset age**	**Age at LP**	**QIgG**	**Qalb**	**IgG index**
NMOSD	Age at LP	0.938**				
	QIgG	0.303**	0.366**			
	Qalb	0.294**	0.335**	0.912**		
	IgG index	0.198#	0.252*	0.514**	0.170	
	CSF-OCB▲	-0.079	-0.061	0.475*	0.158	0.730**
RRMS	Age at LP	0.829**				
	QIgG	0.366**	0.364**			
	Qalb	0.390**	0.428**	0.768**		
	IgG index	0.045	-0.008	0.562**	-0.022	
	CSF-OCB△	-0.161	-0.155	0.293*	0.019	0.532**

**$p<0.01$, * $p<0.05$.

# $p=0.062$

▲Data from 26 patients with AQP4-Ab NMOSD were available.

△Data from 56 patients with RRMS were available.

Abbreviations: NMOSD = neuromyelitis optica spectrum disorder; RRMS = relapsing–remitting multiple sclerosis; LP = lumbar puncture; CSF = cerebrospinal fluid; QIgG = quotient of IgG in CSF and IgG in serum; Qalb = quotient of albumin in CSF and albumin in serum; CSF-OCB = oligoclonal band in CSF.

Supplementary Table S5. Partial correlation analyses between Qalb and diagnosis while controlling the onset age or age at LP.

Control Variables			Qalb
Onset age	Diagnosis	Correlation coefficient *	0.013
		p	0.864
Age at LP	Diagnosis	Correlation coefficient	-0.002
		p	0.983

* A positive value means the variable was associated with NMOSD while a negative one indicates that the variable was correlated with RRMS.

Abbreviations: LP = lumbar puncture; Qalb = quotient of albumin in CSF and albumin in serum.
